# Antibiotic-loaded calcium sulfate beads in spinal surgery for patients with spondylodiscitis: a clinical retrospective study

**DOI:** 10.1186/s12891-022-05230-y

**Published:** 2022-03-19

**Authors:** Xiaojie Tang, Jianyi Li, Chunxiao Wang, Fang Liu, Jianwei Guo, Jiangwei Tan, Qinyong Song, Haifei Cao, Yao Zhang

**Affiliations:** 1 Department of Orthopedics, Yantai Affiliated Hospital of Binzhou Medical University, 717 Jinbu Street, Muping District, Yantai, 264000 Shandong Province People’s Republic of China; 2 Department of Orthopedics, The Affiliated Hospital of Qingdao University, 16 Jiangsu Road, Qingdao, 266003 Shandong Province People’s Republic of China

**Keywords:** Spondylodiscitis, Antibiotic-Loaded, Calcium Sulfate Beads, Spinal Surgery

## Abstract

**Background:**

Various surgical techniques for treating spondylodiscitis have been proposed, but the optimal surgical treatment remains controversial. In this study, we propose a new procedure that is implanting antibiotic-loaded calcium sulfate (CS) beads into the disc after debridement using the Quadrant channel combined with percutaneous fixation through a single-stage posterolateral approach for the treatment of spondylodiscitis. Thus, the purpose of this study is to assess the safety and efficacy of this procedure.

**Methods:**

This study collected the data of 32 patients with spine spondylodiscitis and was surgically treated in our department from July 2015 to August 2020. The Demographic data included age, gender, involved segment, and complications were collected. The intra-operative details, results of culture, functional outcome, radiologic outcome, and length of hospital stay, laboratory examination were recorded.

**Results:**

The mean age of the 32 patients was 61.1 ± 9.7 years old. The mean operative time was 135.0 ± 30.6 minutes, and the mean blood loss was 243.4 ± 92.1 ml. The positive rate of culture was 72%. The mean Visual analogue scale (VAS) and Oswestry Disability Index (ODI) score significantly improved from 7.5 to 1.6 and from 65% to 10%. Cobb angle was significantly improved and could be maintained at final follow-up. New bone formation was observed in all patients. There were no recurrences of infection in our study.

**Conclusions:**

The posterolateral debridement and percutaneous fixation combined with antibiotic-loaded calcium sulfate beads filling are effective in the treatment of spondylodiscitis in terms of infection control, early mobilization, and recovery.

## Background

Spondylodiscitis is an uncommon but potentially life-threatening bacterial infection involving the intervertebral disc and adjacent vertebrae [1]. The annual incidence of spondylodiscitis ranges from 0.5 to 2.2/100,000 inhabitants in Europe [2, 3], and the infection commonly tends to affect the elderly or chronically debilitated patients aged over 50 years [4]. Spondylodiscitis shows mortality rates from 2% to 20% and a morbidity rate of more than 7 % [5]. It can be managed either by non-operative measures, including intravenous/oral antibiotics and immobilization, or by surgical intervention [6]. Surgery for spondylodiscitis is indicated for instability, deformity, neurological deficits, unbearable pain, or disease progression [7]. The surgical goal in spondylodiscitis is to debride the infection, identify and reduce the pathogens, stabilize the deformed and unstable segments, and decompress the neural structures [8].

Anterior, posterior and combined approaches have been used for the treatment of spondylodiscitis [9–16]. However, the optimal surgical treatment remains controversial. Antibiotic-loaded calcium sulfate is widely used in osteomyelitis as a filler of bone defects, but there were only a few reports specifically on antibiotic-loaded carriers to our knowledge [17]. The objective of this retrospective study was to verify the efficiency of antibiotic-loaded calcium sulfate beads applied in a single-stage posterolateral approach for the treatment of spondylodiscitis.

## Materials and methods

Thirty-two consecutive patients who underwent surgical treatment for lumbar/ thoracic spondylodiscitis at our department from July of 2015 to August of 2020 were retrospectively investigated. All the patients were treated with the proposed procedure. Patients with neurological deficits, instability, progressive pain, or progression on magnetic resonance imaging (MRI)/ computed tomography (CT) despite conservative treatment underwent surgical treatment. Demographic data, including age, gender, involved segment, clinical outcomes and complications were recorded (Table 1). The intra-operative details, results of culture, functional outcome, radiologic outcome, blood loss, ASIA classification, and length of hospital stay were recorded (Table 2). The sagittal deformity was evaluated by Cobb’s methods, which is the angulation of the upper and lower endplates of the collapsed vertebral bodies. The Cobb angle, Visual analogue scale (VAS), Oswestry Disability Index (ODI) at the time of initial diagnosis, the 7th post-operative day, and the last follow-up were compared (Table 3). Radiological investigations such as X-rays and computed tomography (CT) were done to document endplate erosion, cavitation, reduction in disc space, and instability, and magnetic resonance imaging (MRI) was serially done for evaluation of response to treatment pre-op and post-op routinely. Clinical evaluation was performed using the ASIA classification, ODI and VAS. The functional outcome of the study was measured by the modified criteria of Kirkaldy–Willis. All the patients were treated by empirical antibiotic therapy or antibiotics according to the results of microbial culture and sensitivity when available. Patients were treated with intravenous broad spectrum antibiotics for 6-8 weeks followed by oral antibiotics for a total period of 3 months. The antibiotic therapy was withdrawn based on clinical improvements and the infection related laboratory test results, such as the average levels of C-reactive protein (CRP) and erythrocyte sedimentation rate (ESR).

**Table 1:** Clinical data of patients

Parameter	Value
Gender	
Female	18
Male	14
Age, average, y	61.1 ± 9.7
Affected levels	
Thoracic	8
Thoracolumbar	4
Lumbar/lumbosacral	20
Concurrent disease	
Diabetes	11
Rheumatism	1
Nephritis	1
Osteoporosis	5
ASIA classification (pre-op)	
C	3
D	16
E	13

**Table 2:** Summary of treatment outcomes, mean ± SD

Parameters	Measurements
Operation time, min	135.0 ± 30.6
Blood loss, mL	243.4 ± 92.1
Hospitalization, d	52.3 ± 10.0
Duration of follow-up, m	25.2 ± 8.1
ASIA classification (post-op)	
C	0
D	3
E	29

**Table 3:** Laboratory findings and VAS、ODI、Cobb angle‾x ± s

	ESR (mm/h)	CRP (mg/L)	VAS	ODI	Cobb angle(°)
Pre-op	82.37 ± 27.11	78.19 ± 27.48	7.50 ± 1.16	65.41 ± 13.46	16.44 ± 4.15
Post-op	53.88 ± 19.89	44.22 ± 17.19	4.44 ± 1.48	27.59 ± 9.64	19.84 ± 5.14
FFU	19.06 ± 8.21	8.12 ± 5.12	1.66 ± 0.65	10.03 ± 5.08	19.25 ± 4.97
F	131.427	122.721	225.452	351.766	54.186
P	<0.001	<0.001	<0.001	<0.001	<0.001

*Pre-op* Pre-operative, *Post-op* Post-operative, *FFU* Final follow-up

### Inclusion criteria

Patients who had one or more of the following characteristics were included in this study: (1) neurological deficit caused by spinal canal involvement, (2) spinal instability caused by large vertebral body destruction, (3) kyphosis observed on imaging, (4) epidural or paravertebral abscess formation, and (5) aged patients could not bear long time bed rest.

### Exclusion criteria

Patients with the following conditions were excluded in this study: (1) the affected vertebral body collapse greater than 1/2 of its total height, (2) follow-up period less than 1 years, and (3) poor general condition made the procedure impossible.

### Operative procedure

After general anesthesia, patients were carefully placed in a prone position, and the surgery was performed through a posterolateral approach. The anteroposterior (AP) view was then used to identify the affected segment and the location of pedicle with fluoroscopy. A 1-2 cm incision is proposed and marked just lateral to the lateral border of the pedicle. The entry point is usually selected to be at or just lateral to the lateral border of the pedicle in the AP view. Instrumentation with percutaneous pedicle screw placement into the infected vertebra and the adjacent vertebra above and below the infected segment was performed on the side with less infection involvement.

**Fig. 1:**
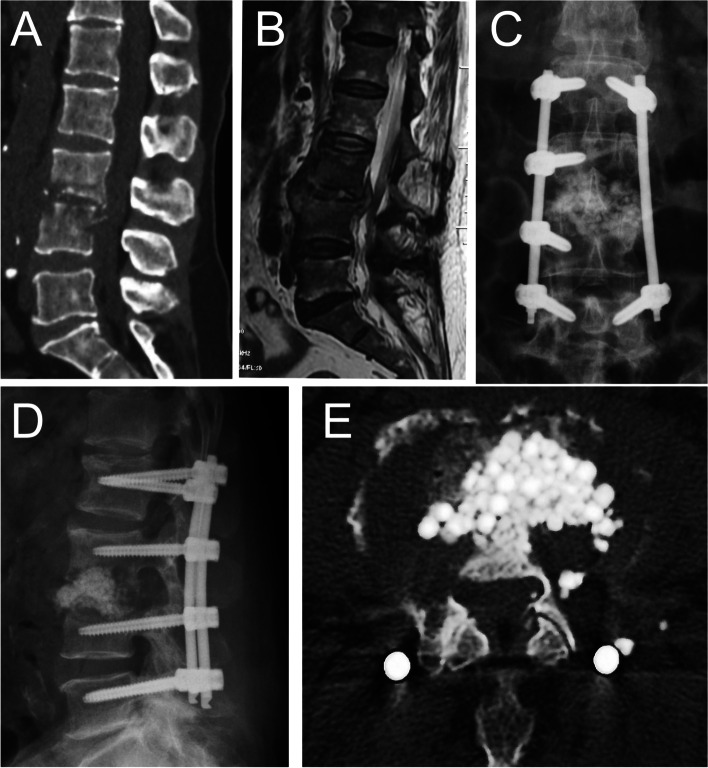
A 65-year-old female suffered from low back pain with lumbar spondylodiscitis. **A** Preoperative sagittal CT showed L3/4 disc involvement and bone defects. **B** MRI showed abscess formation which involves the spinal canal. **C,D** Postoperative anteroposterior and lateral radiographs after short segmental fixation showed good spinal alignment. **E** Postoperative axial CT showed the intervertebral space is filled with antibiotic-loaded calcium sulfate beads

**Fig. 2:**
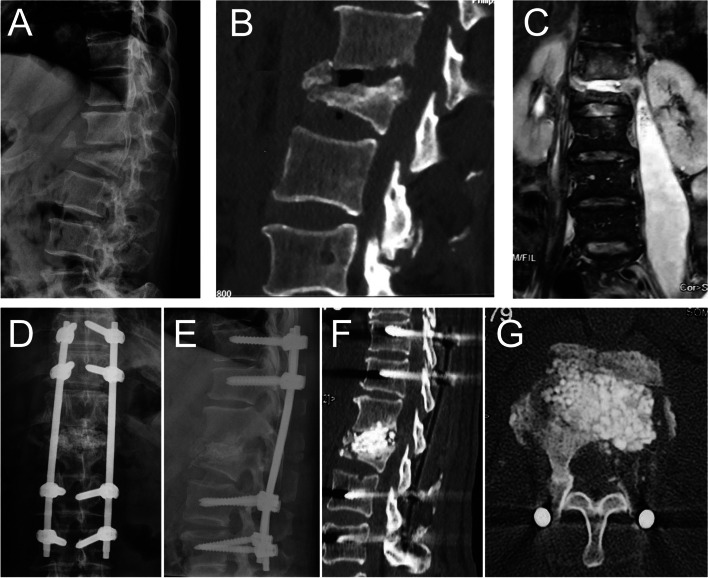
A 66-year-old male with thoracolumbar spondylodiscitis. **A,B** Preoperative sagittal X-ray and CT showed the collapse of the vertebral body, leading to the kyphosis. **C** Preoperative coronal MRI showed psoas abscess formation. **D,E** Postoperative anteroposterior and lateral radiographs after long segmental fixation showed acceptable spinal alignment. **F,G** Postoperative sagittal and axial CT showed the antibiotic-loaded calcium sulfate beads in the disc space

**Fig. 3:**
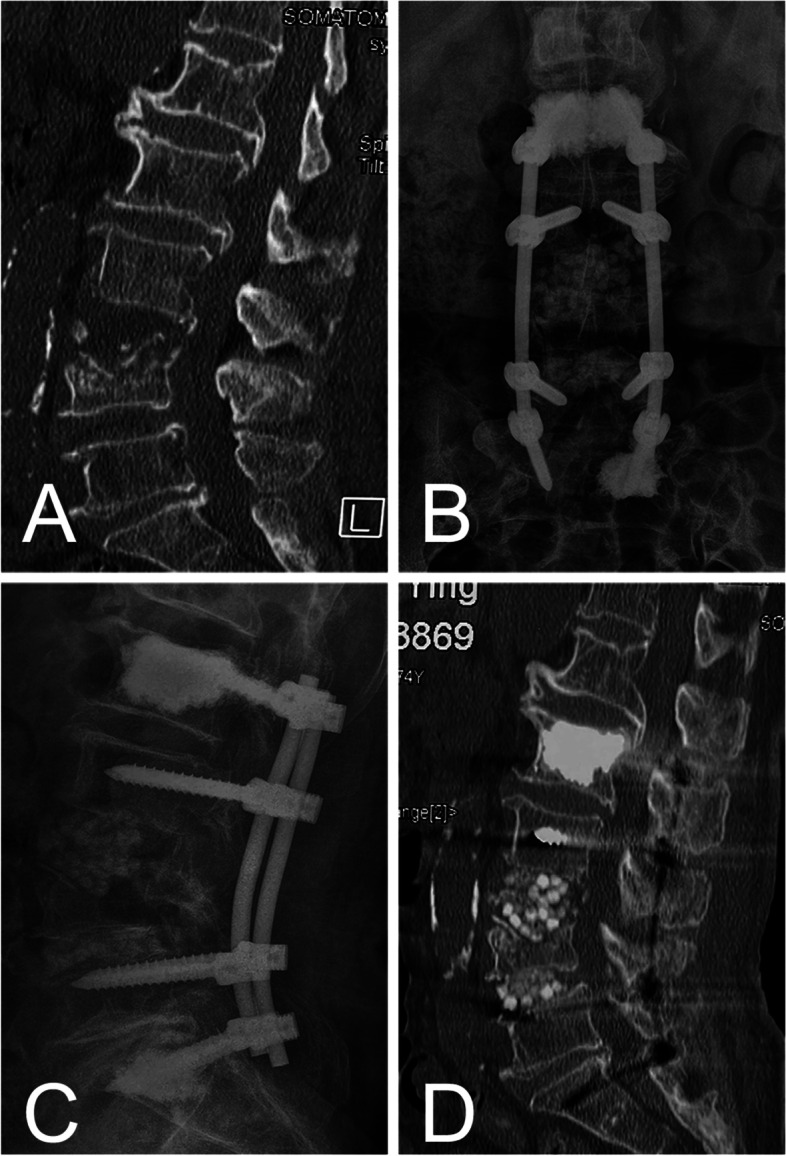
A 74-year-old female suffered from spondylodiscitis with severe osteoporosis. **A** Preoperative sagittal CT showed severe bone destruction. **B,C,D** Postoperative anteroposterior and lateral radiographs and sagittal CT showed the bone cement enhanced screw fixation and calcium sulfate beads buttressing the anterior column

Pedicle screw was inserted 1 level above and 1 level below the most destroyed vertebra on the opposite side (Fig. 1). For patients with severe vertebral body destruction (but less than 1/2 of the total height), longer segmental fixtion was used to ensure the postoperative stability (Fig. 2). For patients with osteoporosis, cement enhanced screws were used at the end vertebra (Fig. 3). A longitudinal incision around 3 cm in length was made 2 cm beside the midline on the affected side. The skin, subcutaneous tissue, and lumbar fascia were cut in turn, and then the paravertebral muscles were separated by the Quadrant instrument (Medtronic USA) to establish a channel to the infected segment. The lateral edge of the upper lamina and superior articular process were exposed. Kerrison rongeurs were used to remove part of the lateral edge of the upper lamina and superior articular process on the more affected side by the infection in a transforaminal approach. After the exposure of the exit nerve root, the posterior annular fibrosis was cut to probe into the disc, all the pus, the granulomatous tissue, and/or necrotic material were removed from the disc space. Radical debridement was performed using a set of curettes until sclerosing bone was completely removed and healthy, bleeding margins were obtained.

**Fig. 4:**
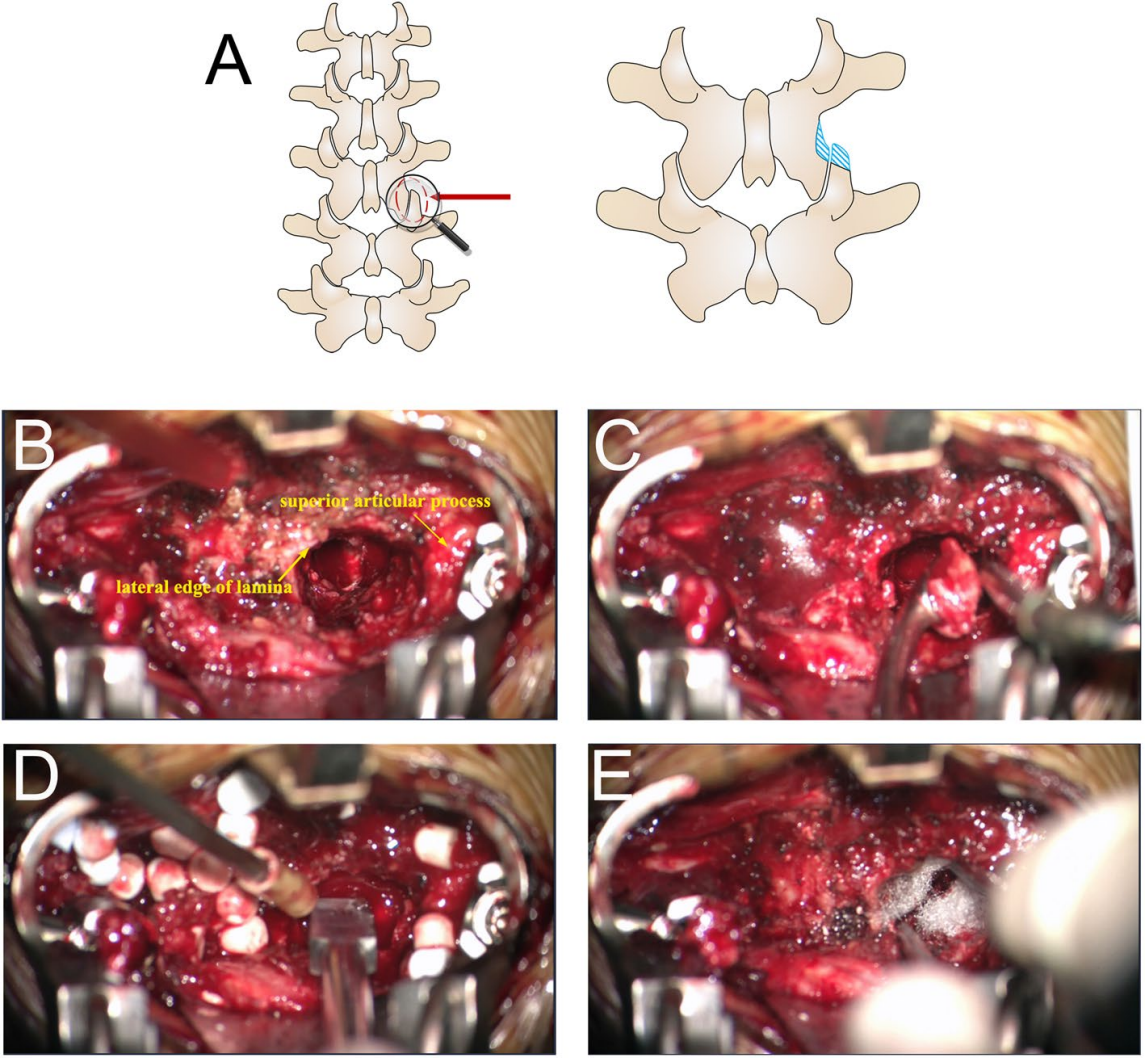
**A** Schematic picture of transforaminal approach for spondylodiscitis. **B** Intraoperative picture of the fenestration. **C** Debridement of the lesion with curette. **D** Insertion of the CS beads. **E** Haemostatic gauze was put between the beads and the canal to prevent later migrating backward

For patients with spinal canal involvement, we go more medially to the canal by resecting more of the anterior part of the superior articular process and for patients with psoas abscess, we go anteriorly to the psoas muscle [18]. The kit containing 5ml calcium sulfate powder (STIMULAN, Biocomposites Ltd, England), 0.5 g of vancomycin hydrochloride (for Gram-positive cocci) /cefoperazone and sulbactam sodium (for Gram-negative bacilli), and 2.5-3 ml sterile water were mixed for 30 s to form a paste, which was pressed into 4.8-mm-diameter hemispherical cavities in a flexible mold. The beads were left undisturbed for 20 min to set. When set, the beads were removed by flexing the mold and implanted into the debrided site to reconstruct the anterior column and control the infection. For patients with spinal canal involvement, the canal had to be re-explored after the filling of the disc space with CS to avoid the beads invasion. Some haemostatic gauze was put between the beads and dura to prevent possible later migration backward (Fig. 4). Finally, posterior instrumentation was finished and the debrided material was routinely sent for histopathologic examination as well as bacterial culture and antibiotic sensitivity testing.

### Statistical analysis

All patients with complete initial data were considered for inclusion in the retrospective analysis. All values are expressed as mean ± SD. Repeated measurement data of continuous numerical variables were analyzed by repeated measurement ANOVA, comparisons between the different time points were analyzed using the student’s paired t-test. All statistical evaluations were performed with SPSS 22.0 (IBM, Armonk, New York). *P* < 0.05 was considered statistically significant.

## Results

The demographic details and patients’ characteristics are presented in table 1. Thirty-two consecutive patients who underwent this surgery for spondylodiscitis had been identified from 2015 to 2020 and their data were investigated. The mean duration of follow-up was 25.2 ± 8.1 months (range 12 to 36 months). The surgical incisions were healed without chronic infection, fistula formation. No recurrences were observed in this group. All patients had significant improvement in constitutional symptoms and back pain after surgery. Early ambulation was permitted the next day after the surgery in all patients. No perioperative complications related to instrumentation or decompression were reported.

The mean patient age was 61.1 ± 9.7 years (range 43–82), with 56 % of the cases being female. The intra-operative amount of blood loss was 243.4 ± 92.1 ml (range 120 to 520 ml) and the duration of surgery was 135.0 ± 30.6 min (around 100 to 240 mins). The majority of the cases involved the lumbar region (62.5%), 25% involving the thoracic spine and 12.5% at the thoracolumbar region. Local tissue cultures for all patients and blood cultures for patients with high fever were performed preoperatively. 14 cases (44%) were Staphylococcus aureus, 5 cases (16%) Escherichia coli, 3 cases (9%) Mycobacterium tuberculosis, 1 case (3%) Propionibacterium acnes, and 9 cases (28%) were culture negative.

**Fig. 5:**
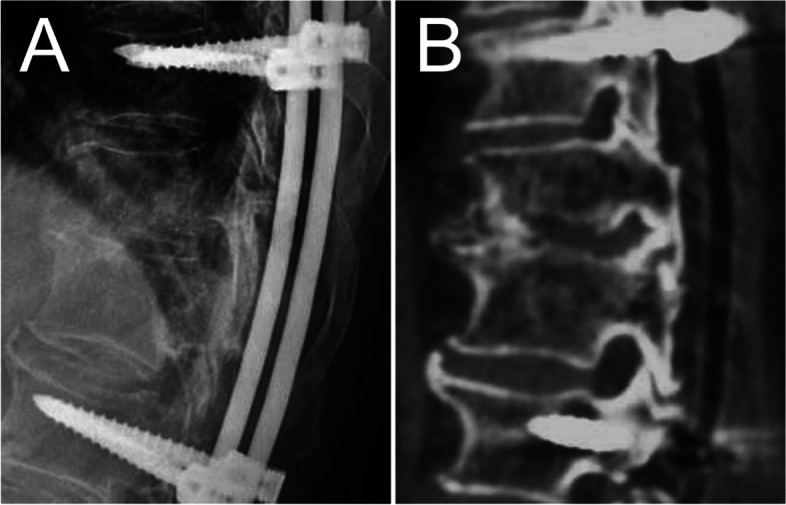
A 72-year-old male 6 months after surgery. **A** Sagittal X-ray showed indistinct bone connection between the affected segments and **B** Sagittal CT reconstruction showed obvious peripheral bone bridge formation

The average Cobb angle was significantly improved at 19.8 ± 5.1° post-op and had resolved to 19.2 ± 4.9° at final follow-up. There were statistically differences between the pre-op Cobb angle and the post-op angle and the angle at the final follow-up (*P*<0.001). The VAS and the ODI values on the seventh-day post-op and at the last follow-up were significantly lower compared with those in pre-op period (*P* < 0.001). Significant differences were found between the post-op and last follow-up. The ESR and CRP returned to normal levels in all patients within 3 months after surgery. The average hospital stay was 52.3 ± 10.0 days. The average time of the new bone formation and peripheral connection in the lesion segment was 4.6±1.1 months (range 3–6 months), judging by plain radiograph and/or CT scan (Fig. 5). At the last follow-up, neurological recovery was observed in all the patients with the previous neurological deficit (Table 2). According to the Kirkaldy-Willis functional criteria, 14 patients were finally evaluated as “excellent”, 17 patients were “good” and one patient was “fair”.

## Discussion

Spondylodiscitis is a most common spinal infection, which affects the vertebral bodies, intervertebral disk, paraspinal tissues, and also the posterior bony elements occasionally [19]. Traditionally, conservative treatment including immobilization and systemic administration of antibiotics is the first choice for pyogenic spondylitis [20, 21]. Surgical treatment is indicated in patients with progressive biomechanical pain, refractoriness to antibiotic therapy, epidural abscesses, neurological impairment or segmental instability [22]. Surgical intervention is recommended as it could relieve pain, maintain vertebral column balance, improve neurologic function, and bring higher quality life [23].

Debridement and reconstruction are two main principles of the surgical treatment of pyogenic spondylitis [24, 25]. The anterior approach provides direct visualization for radical debridement and decompression without affecting the posterior elements, which is the best way to control the infection and promote definitive healing, and it also can warrant adequate and strong reconstruction with modern instrumentation tools [10–12]. However, anterior surgery alone does not adequately correct kyphosis and could not bear physiological loads, the recurrence of kyphosis following it are difficult to treat [9, 26]. To overcome these limitations, some authors advocated posterior spinal stabilization and fusion. The posterior approach could give better kyphotic deformity correction and vertebral stabilization, fewer complications and less surgical invasiveness [16]. Meanwhile, the infected intervertebral discs, vertebral endplates, and vertebral body tissues could be adequately debrided through a posterior-only approach to assure the excellence of the radiographic and clinical results [13, 15]. However, a considerable portion of authors believed that the posterior-only approach has the drawbacks of insufficient removal of infected tissue compared with the anterior approach [27]. Thus a combination of anterior debridement with posterior fixation is widely used for the treatment of lumbar pyogenic spondylodiscitis [14, 28–30]. Nevertheless, the combined approach increases the risk of morbidity associated with the prolonged duration of operation, anesthesia times, the additional blood loss and operative trauma [29]. In our study, all the patients underwent a single-stage posterolateral approach, which is an effective method with easy operation, less invasion and stable secure fixation. The debridement performed with Quadrant system and percutaneous pedicle screw fixation was used to minimize the damage to the paraspinal muscles [18]. The function of the facet joints was preserved well enough to maintain the sagittal stability of the spine by tension band effect instead of cantilever effect if the facet joints were removed. This may be the main reason that in our group the final kyphotic Cobb was maintained well during the follow up period in addition to bone cement enhancement for some osteoporotic patients. Moreover, there was no disturbance to the peritoneum, the abdominal vessels, the psoas muscle, and the lumbosacral nerves in this posterolateral procedure.

Antibiotic-loaded cement (AIBC) is widely used to treat or prevent infections in total hip and knee arthroplasty because it can lead to a locally high antibiotic concentration [31]. Nonetheless, the burst release of antibiotics and microbial colonization of the non-degradable cement has led to an advanced investigation for more antibiotic delivery [32]. Calcium sulphate has a long history as antibiotic carrier material, which is a resorbable osteoconductive scaffolds [33]. It also can be mixed with heat-sensitive antibiotics because there is very little temperature rise on curing [17, 34]. The mechanical strength of CS is comparable to cancellous bone and is hydrolyzed slowly in bone, lasting for about 6–12 weeks [35]. The antibiotic-loaded beads demonstrated high bioactivity in preventing and eliminating the residual bacteria, with long periods of sustained efficacy. It's a good solution for the shortcoming of insufficient removal of infected tissue in the posterior procedure. The cement beads could be absorbed completely in about 8-12 weeks after surgery, and new bone gradually formed while antibiotics were released to control the infection. The imaging examination showed the infected site healing well with no recurrence during the follow-up in our series. Percutaneous pedicle screw instrumentation was used to avoid unnecessary muscle dissection and tissue disruption, decrease blood loss and complications, provide immediate stability [36]. Meanwhile, pedicle screws were inserted into the infected and adjacent vertebra to avoid decreasing mechanical stability by longer fixation levels [37]. However, we used longer segmental fixation in patients with severe vertebral body destruction, which made the screw insertion into the infected vertebral body impossible, to ensure postoperative stability. Bone cement enhanced screws were applied to obtain immediate stability after surgery for patients with osteoporosis. With these techniques, all patients could ambulate the second day after surgery.

Antibiotics targeted toward the causative pathogen appear to be the most important factor to determine the success rate of treatment. Identifying the causative pathogens is one of the key factors to achieve cure in our studies. A positive culture was obtained in 23 patients (72%) while Staphylococcus aureus is the most frequent pathogen (73%) in our studies. Systemic antibiotic treatment duration ranged from 6 weeks to 12 weeks, which was consistent with the previous literature description [23]. Appropriate antibiotic therapy based on the causative pathogen is crucial and can lead to good clinical results. After sending blood culture and disc space aspiration, all patients immediately started empirical intravenous antimicrobial therapy. Then, an appropriate antibiotic was administrated according to the results of microbial culture and sensitivity [38].

This study is mainly aimed at patients with bone defects causing spinal instability, kyphosis, or progressive neurological impairment. Percutaneous fixation after the posterolateral debridement could maintain the stability of the spine and correct kyphosis, which could relieve the severe pain of the patients and make early ambulation possible. Our data showed the pain was significantly relieved and the ODI index was significantly increased after surgery, all patients could return to normal life. Internal local immobilization also helps control the infection spreading and make the general recovery earlier. The patients’ ESR and CRP levels decreased significantly 7 days after surgery and returned to normal in a mean of 4 weeks after surgery, indicating that the inflammation was well controlled. The CS antibiotic bone granules were absorbed in 2 to 3 months gradually, and the bony bridge was observed in all patients in a mean of 4.1 months after surgery. The Cobb Angle was maintained at the final follow-up with no kyphotic deformity aggravated compared with the post-op. All the 20 patients with preoperative dysfunction recovered to different degrees after operation, and the excellent and good rate reached to 94.4%.

However, this study has some limitations. The current study is a small-scale retrospective data analysis in a single institution with no control group. We need a prospective controlled study in a large number of patients to more powerfully identify the effectiveness of this technique in future.

## Conclusions

Most patients improved significantly not only in their general conditions but also in their neurological status after surgery without any serious complications. The posterolateral debridement and percutaneous fixation combined with antibiotic-loaded calcium sulfate beads filling is a safe and effective procedure for spondylodiscitis in terms of infection control, early mobilization and recovery.

## Data Availability

The data used and analyzed during the current study are available in anonymized form the corresponding author on reasonable request.

## Abbreviations

CS: Calcium sulfate
CT: Computed tomography
MRI: Magnetic resonance imaging
VAS: Visual analogue scale
ODI: Oswestry Disability Index
Pre-op: Pre-operative
Post-op: Post-operative
FFU: Final follow-up
ESR: Erythrocyte sedimentation rate
CRP: C-reactive protein
